# RKIP Inhibition in Cervical Cancer Is Associated with Higher Tumor Aggressive Behavior and Resistance to Cisplatin Therapy

**DOI:** 10.1371/journal.pone.0059104

**Published:** 2013-03-19

**Authors:** Olga Martinho, Filipe Pinto, Sara Granja, Vera Miranda-Gonçalves, Marise A. R. Moreira, Luis F. J. Ribeiro, Celso di Loreto, Marsha R. Rosner, Adhemar Longatto-Filho, Rui Manuel Reis

**Affiliations:** 1 Life and Health Sciences Research Institute (ICVS), Health Sciences School, University of Minho, Braga, Portugal; 2 ICVS/3B’s - PT Government Associate Laboratory, Braga/Guimarães, Portugal; 3 Molecular Oncology Research Center, Barretos Cancer Hospital, Barretos, São Paulo, Brazil; 4 Department of Pathology of the School of Medicine of the Federal University of Goias, Goiânia, Goias, Brazil; 5 Hospital Araújo Jorge, Goiânia, Goias, Brazil; 6 Pathology Division, Adolfo Lutz Institute São Paulo, São Paulo, Brazil; 7 Ben May Department for Cancer Research, University of Chicago, Chicago, Illinois, United States of America; 8 Laboratory of Medical Investigation (LIM) 14, Faculty of Medicine, São Paulo, São Paulo, Brazil; Ospedale Pediatrico Bambino Gesú, Italy

## Abstract

Cervical cancer is one of the most common cancers in women worldwide, being high-risk group the HPV infected, the leading etiological factor. The raf kinase inhibitory protein (RKIP) has been associated with tumor progression and metastasis in several human neoplasms, however its role on cervical cancer is unclear. In the present study, 259 uterine cervix tissues, including cervicitis, cervical intraepithelial lesions and carcinomas, were analyzed for RKIP expression by immunohistochemistry. We found that RKIP expression was significantly decreased during malignant progression, being highly expressed in non-neoplastic tissues (54% of the samples; 73/135), and expressed at low levels in the cervix invasive carcinomas (∼15% (19/124). Following *in vitro* downregulation of RKIP, we observed a viability and proliferative advantage of RKIP-inhibited cells over time, which was associated with an altered cell cycle distribution and higher colony number in a colony formation assay. An *in vitro* wound healing assay showed that RKIP abrogation is associated with increased migratory capability. RKIP downregulation was also associated with an increased vascularization of the tumors *in vivo* using a CAM assay. Furthermore, RKIP inhibition induced cervical cancer cells apoptotic resistance to cisplatin treatment. In conclusion, we described that RKIP protein is significantly depleted during the malignant progression of cervical tumors. Despite the lack of association with patient clinical outcome, we demonstrate, *in vitro* and *in vivo*, that loss of RKIP expression can be one of the factors that are behind the aggressiveness, malignant progression and chemotherapy resistance of cervical cancer.

## Introduction

Cervical cancer is the third most commonly diagnosed cancer and the fourth leading cause of cancer death in females worldwide, accounting for 9% (529.800) of the total new cancer cases and 8% (275.100) of the total cancer deaths among females in 2008 [1]. Persistent infection with high-risk types of human papillomavirus (HPV) is a *sine-qua-non* condition for cervical cancer development. HPVs infect epithelial cells and cause a variety of lesions ranging from common warts to cervical neoplasia and cancer [2]–[4]. Tumors of the cervix are divided into three different histological subtypes: Uterine squamous cell carcinomas (SCC) is the most frequent, followed by adenocarcinoma (AC) and adenosquamous carcinoma (ASC), which is an uncommon subtype [5]. HPV infection alone is not enough for triggering cervical cancer and HPV-mediated oncogenesis also requires the accumulation of additional genetic changes that occur over time following initial infection [6]. It may take several years for an *in situ* neoplasm to progress to an invasive carcinoma. The mechanism of clonal evolution, which involves the selection of cells with invasive or metastatic potential, also remains unsolved.

Raf kinase inhibitory protein (RKIP; also known as PEBP1, for phosphatidylethanolamine-binding protein1), as indicated by the name, was first identified as the endogenous inhibitor of the RAF/MEK/ERK pathway, inhibiting Raf-1 activation [7]–[9]. Actually, RKIP has been implicated in various intracellular signaling pathways that control cell growth [10], [11], motility [12], [13], epithelial to mesenchymal transition (EMT) [14] and differentiation [15]. RKIP is widely expressed in normal human tissues, highlighting its role in various physiologic processes [16], but is considered to be a metastasis suppressor in cancer [17], being its loss or reduced expression associated with malignancy and prognosis in many types of metastatic and aggressive cancers [10], [11], [18]–[34].

A previous study, done in a small fraction of patients, found by expression microarray analysis that RKIP is one of the genes that is differentially expressed between tumor samples from cervical cancer patients with or without lymph node metastasis [35]. More recently, it was found in a large series of patients that RKIP protein is significantly downregulated in cervical cancer and lymph node metastasis [36]. Additionally, another study with HeLa cervical cancer cells showed that RKIP, through regulation of the ERK pathway, has an important role in mitotic checkpoint regulation [37]. Hence, the previous findings, prompted us to elucidate the biological role of RKIP in cervical cancer malignant progression and chemotherapy response. Therefore, we first assessed the expression levels of RKIP protein in both non-malignant and tumoral cervical samples, and assessed its role in the clinical outcome of cervical cancer patients. Secondly, we assessed *in vitro* and *in vivo* the biological function of RKIP downregulation in cervical cancer malignancy and chemotherapy response.

## Materials and Methods

### Tissue Samples

For the present work, 259 cervical tissues were analyzed, which included 45 cervicitis, 47 low-grade squamous intraepithelial lesions (LSIL), 43 high-grade squamous intraepithelial lesions (HSIL), 70 squamous cell carcinomas (SCC), 41 adenocarcinomas (AC) and 13 adenosquamous carcinomas (ASC) (Table 1). The paraffin samples containing the cervical non-malignant lesions were retrieved from the files of the Pathology division of Adolfo Lutz Institute São Paulo, Brazil, and the invasive cervical tumor samples were retrieved from Araújo Jorge Hospital and School of Medicine of the Federal University of Goiás (Goiânia, Goias State, Brazil). All histopathological diagnoses were reviewed by the authors and categorized according to the WHO classification. All patients with cervical cancer were females of Brazilian origin, with a mean age of 49 years (range 23–74 years). Follow-up data was available for 72 patients, and collected through direct interview with patients or their relatives, and by review of in-hospital patient files. The median follow-up time (overall survival) was 35.5±42.0 months (range, 2–144 months).

**Table 1:** RKIP expression in cervical lesions.

			RKIP Expression		
Type of Lesion		N	Negative (%)	Positive (%)	*p*
**Cervicitis**		45	16 (35.6)	29 (64.5)	
**LSIL**					
	**CIN1**	47	26 (55.3)	21 (44.7)	
**HSIL**					
	**CIN2**	16	6 (37.5)	10 (62.5)	
	**CIN3**	27	14 (51.9)	13 (48.1)	
**LSIL**		47	26 (55.3)	21 (44.7)	0.404
**HSIL**		43	20 (46.5)	23 (53.5)	
**Invasive Carcinoma**					
	**AC**	40	33 (80.5)	8 (19.5)	0.643
	**SCC**	70	61 (87.1)	9 (12.9)	
	**ASC**	13	11 (84.6)	2 (15.4)	
**Cervicitis**		45	16 (35.6)	29 (64.5)	0.087
**SIL**		90	46 (51.1)	44 (48.9)	
**Benign Lesions**		135	62 (46.0)	73 (54.0)	<0.001
**Cervical Cancer**		124	105 (84.7)	19 (15.3)	

Cervical intraepithelial neoplasia (CIN); Low-grade squamous intraepithelial lesions (LSIL); high-grade squamous intraepithelial lesions (HSIL); squamous cell carcinomas (SCC); adenocarcinomas (AC); adenosquamous carcinomas (ASC).

All the samples enrolled in the present study were unlinked and unidentified from their donors. Due the retrospective nature of the study, no written informed consent from patients was obtained. The Local Ethical Review Committees of the involved institutions (Ethics committee in Human Research of Adolfo Lutz Institute São Paulo and of Araújo Jorge Hospital from School of Medicine of the Federal University of Goiás, Brazil) approved the work and waived the need for written informed consent.

### Cell Lines

For the present study it were used three cervical carcinoma cell lines: HeLa cell line, kindly provided by Dr^a^ Elsa Logarinho (IBMC, Portugal) [38], SiHa and C-33A cell lines that were kindly provided by Dr^a^ Luisa Villa (INCT-HPV, Brazil) [39]. All the cell lines were grown and maintained at 37°C and 5% CO_2_ in Dulbecco’s Modified Eagle’s Medium (DMEM 1×, High Glucose; Gibco, Invitrogen) supplemented with 10% Fetal Bovine Serum (FBS; Gibco, Invitrogen) and 1% penicillin/streptomycin solution (Gibco, Invitrogen) (DMEM-10).

### Drugs

Cisplatin (cis-Diammineplatinum(II) dichloride) was obtained from Sigma-Aldrich and diluted in 0.9% NaCl for a stock solution of 10 mM. The drug was subsequently prepared as intermediated dilutions to obtain an equal quantity of drug vehicle (1% final concentration) in each of the conditions studied. In all experimental conditions the drugs were diluted in 0.5% FBS culture medium (DMEM-0.5).

### Immunohistochemistry and Immunocytochemistry Analysis of RKIP

Histological slides with 4 µm-thick tissue sections were subjected to immunohistochemical analysis according to the streptavidin-biotin peroxidase complex system (UltraVision Large Volume Detection System Anti-Polyvalent, HRP; LabVision Corporation), using the primary antibody raised against RKIP (Millipore, reference 07–137) diluted 1∶600, incubation 1H at RT, as previously described [18], [25], [40], [41].

Sections were scored double-blind for cytoplasmatic expression following a semi-quantitative criterion: (*−*), 0% of immunoreactive cells; (+), *<*5% of immunoreactive cells; (++), 5–50% of immunoreactive cells; and (+++), *>*50% of immunoreactive cells. Samples with scores (*−*) and (+) were considered negative, and those with scores (++) and (+++) were considered positive.

For RKIP immunocytochemical analysis of cervical carcinoma cell lines, the cells were plated on glass coverslips placed into 12-well plates, and allowed to adhere overnight. The immunocytochemistry procedure was performed as previously described [41].

### Generation of shRKIP Stably Expressing Cell Lines

For generation of cell lines stably expressing shRKIP, we used the PQY15 vector, containing a 19 bp shRNA for RKIP, as previously described [37], [41], [42]. The transfection was done by using the FUGENE HD reagent (Roche) as recommended by the manufacturer, with 2 µg of plasmid at a ratio of 6∶2 (Reagent:Plasmid). The cells (2×10^5^) were plated onto a 12-well plate until 80% of confluence and transfected in DMEM medium, without FBS or antibiotics addition, during 24 hours [41]. After that, stable transfectants were selected with 0.5 µg/ml (HeLa) or 2 µg/ml (C-33A and SiHa) of puromycin in DMEM-10. The empty vector was also transfected as a control.

### Western Blot Analysis

The cells were plate in a 6 well plate at a density of 8×10^5^ cells per well and allowed to adhere at least 24 hours. The cells were serum starved for 6 hours before protein isolation. When necessary, the cells were also stimulated with 10 ng/ml of EGF by 10 minutes before the end of the 6 hours of starvation. Also, for cisplatin experiments the cells were exposed to various concentrations of cisplatin for an additional period of 24 hours in DMEM-0.5. The cells were scraped in cold PBS and lysed in buffer containing 50 mM Tris pH 7.6–8, 150 mM NaCl, 5 mM EDTA, 1 mM Na3VO4, 10 mM NaF, 10 mM NaPyrophosphate, 1% NP-40 and 1/7 of Protease cocktail inhibitors (Roche). Western blotting was done using standard 12% SDS-PAGE gel, loading 20 µg of protein per lane, with detection by enhanced chemiluminescence (SuperSignal West Femto Maximum Sensitivity Substrate, Pierce). RKIP expression was measured using a specific antibody against RKIP (dilution 1∶2000, Upstate Biotechnology). Activated ERK and EGFR were assessed using the antibody phospho-p44/42 MAPK (Thr202/Tyr204) and phospho-EGFR (Y1068) (dilution 1∶1000, Cell Signaling Technology). The total form of ERK and EGFR were also assessed with the antibodies p44/42 MAPK (Erk1/2) (clone 137F5, dilution 1∶1000, Cell Signaling Technology) and EGFR (dilution 1∶500, clone 31G7, ZYMED Laboratories). For apoptosis assessment it were used antibodies against cleaved PARP (clone Asp214, dilution 1∶1000, Cell Signaling Technology), cleaved caspase-9 (clone Asp330, dilution 1∶1000, Cell Signaling Technology) and PARP (dilution 1∶1000, Cell Signaling Technology). For loading control we used β-Tubulin (clone AA2, dilution 1∶5000, Upstate Millipore). All the primary antibodies were incubated for one hour at RT.

Blots detection was done by chemiluminescence (ECL Western Blotting Detection Reagents, RPN2109, GE Healthcare) in ImageQuant™ LAS 4000 mini (GE Healthcare) or using X100 Hyperfilm ECL (Amersham, GE Healthcare).

### Cell Viability and Proliferation Assays

The cells were plated into 96-well plates in triplicate at a density of 3×10^3^ cells per well and allowed to adhere overnight in DMEM-10. After 6 hours of serum starvation the viable or proliferative cells were quantified using the Cell Titer96 Aqueous cell proliferation assay (Promega) or with Cell Proliferation ELISA, BrdU (colorimetric, Roche Applied Science) and used for the time 0 value. Then, cells were incubated in DMEM medium without serum during 24, 48 and 72 hours and cell viability and proliferation was again assessed by Cell Titer96 Aqueous cell proliferation assay and Cell Proliferation ELISA, BrdU (colorimetric), respectively. The results were calibrated to the starting value (time 0 h, considered as 100% of viability/proliferation) and expressed as the mean ± SD. The assay was done in triplicate at least three times.

To determine the concentration at which 50% of the cell growth is inhibited by cisplatin treatment (IC_50_ concentration), the cells were plated into 96-well plates at a density of 3×10^3^–5×10^3^ cells per well and allowed to adhere overnight in DMEM-10. Subsequently, the cells were treated with increasing concentrations of the drug diluted in DMEM-0.5. After 48 hours, cell viability was quantified using Cell Titer96 Aqueous cell proliferation assay (MTS) (Promega). The results were expressed as mean ± SD viable cells relatively to drug vehicle alone (considered as 100% viability). The IC_50_ concentration was calculated by nonlinear regression analysis using GraphPad Prism software.

### Wound Healing Migration Assay

The cells were seeded in 6-well plates and cultured to at least 95% of confluence. Monolayer cells were washed with PBS and scraped with a plastic 200 µL pipette tip and then incubated with fresh DMEM medium without serum. The “wounded” areas were photographed by phase contrast microscopy at 12, 24 or 48 hours’ time points. The relative migration distance was calculated by the following formula: percentage of wound closure (%) = 100 (A–B)/A, where A is the width of cell wounds before incubation, and B is the width of cell wounds after incubation [41]. Results are expressed as the mean ± SD. The assay was done in triplicate at least three times.

### Cell Cycle Analysis

The cells were plated in a 6-well plate at a density of 2×10^5^ cells per well and allowed to adhere overnight. After 6 hours of serum starvation the cells were incubated with fresh DMEM medium without serum during 24 hours. Cells were tripsinized and fixed in 70% ethanol at least 30 minutes and then stained during 1 hour at 50°C with a propidium iodide (PI) solution (20 µg/mL of PI and 250 µg/mL of RNAse in a solution of 0.1% Triton X-100 in PBS). Cell cycle analysis of the PI stained cells was performed by flow cytometry (LSRII, BD Biosciences). The percentage of cells in each phase of the cell cycle was determined with the software FlowJo version 7.6.3. The results were expressed as the mean ± SD of the percentage of cells in G1 phase or G2/M plus S phase [41]. The assay was done in triplicate at least three times.

### Soft Agar Colony Formation Assay

The soft-agar colony formation assay was done using standard methods, as previously described by us [43]. Briefly, 1 mL underlayers (base agar layers) consisting of 0.6% agar medium were prepared in 6-well plates by combining equal volumes of 1.2% Noble agar with either 2× DMEM medium with 20% FBS. The cells were trypsinized, centrifuged, and ressuspended in 0.35% agar medium (top agar layer; equal volumes of 0.7% Noble agar and 2× DMEM with 20% FBS); 2×10^3^ cells were then plated onto the previously prepared base agar layers. The cells were incubated at 37°C in a humidified 5% CO_2_ atmosphere for 15 days and the colonies formed stained with 0.05% violet crystal for 15 minutes. Stained colonies were photographed using a stereomicroscope (Olympus S2×16) and a digital camera (Olympus DP71) and counted with Image J Software. Only colonies with a size greater than 775 µm^2^ (approximately 31.4 µm of diameter) were counted [43]. Results are expressed as the mean ± SD. The assay was done in triplicate at least three times.

### Chick Chorioallantoic Membrane (CAM) Assay

To assess *in vivo* tumor proliferation and angiogenesis we used the CAM assay as previously described [41], [43]. Fertilized chicken eggs were incubated at 37°C and 70% humidity, and on day 3 of development, a window was made into the shell, which was sealed with tape, and the eggs were returned to the incubator. On day 9 of development, small plastic rings were placed on the CAM and on day 10 of development 3×10^6^ cells, resuspended in 20 µl of DMEM medium, were injected in the rings over the CAM. On day 17 of development, the tumor formed was photographed *in ovo* using a stereomicroscope (Olympus S2×16), and the perimeter of the tumor was measured using Cell B software (Olympus). Results are expressed as the mean ± SD.

Finally, the chicken eggs were sacrificed at −80°C for 10 minutes, and CAM and tumors were fixed with paraformaldehyde at 4% and photographed *ex ovo* for blood vessels counting. The tumors were embedded in paraffin and processed for histological analysis.

### Statistical Analysis

Correlations between RKIP expression and clinical data from patients were performed using the chi-square test (χ2-test). Cumulative survival probabilities were calculated using the Kaplan-Meier method. Differences between survival rates were tested with the log-rank test. The statistical analysis was performed using SPSS software for Windows, version 17.0. For *in vitro* and *in vivo* assays, single comparisons between the different conditions studied were done using Student’s t test, and differences between groups were tested using two-way analysis of variance (ANOVA). Statistical analysis was done using Graph Pad Prism version 5. The level of significance in all the statistical analysis was set at p<0.05.

## Results

### RKIP Expression in Cervical Tissues

**Figure 1:**
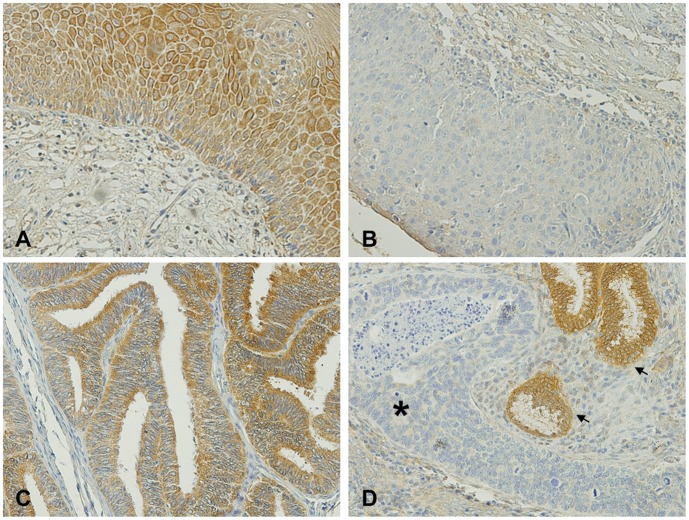
Immunohistochemistry analysis of RKIP in cervical tissues. **A)** Positive expression in a cervicitis. **B)** High-grade squamous intraepithelial lesion with negative expression. **C)** Adenocarcinoma tissue depicting positive expression. **D)** Negative adenocarcinoma (*) with adjacent normal cells (arrow) depicting positive staining. All the pictures were taken with at 200× magnification.

An immunohistochemistry approach was done to detect RKIP protein expression and distribution in cervical lesions (Fig. 1).

Cytoplasmatic positive expression of RKIP was observed in 64.5% (29/45) of the cervicitis samples, in 44.7% (21/47) of LSIL and 53.5% (23/43) of HSIL (Table 1) (Fig. 1A and B). No significant differences were observed in the expression levels of RKIP between LSIL and HSIL (*p* = 0.404), and also between cervicitis and squamous intraepithelial lesions (SIL) in general (*p* = 0.087), Table 1). Concerning malignant lesions, RKIP was present at low levels, with only 19.5% (8/41) of AC, 12.9% (9/70) of SCC and 15.4% (2/13) of ASC depicting positive staining (Table 1) (Fig. 1C and D). No significant differences were observed between RKIP expression in the different malignant lesions studied (*p* = 0.643, Table 1). However, when grouping the benign lesions (cervicitis and SIL) and the malignant lesions (AC, SCC and ASC), a statistical significant decrease of RKIP expression was observed in cervical cancer samples (*p*<0.001) when compared with the benign lesions (Table 1).

No correlations were found between RKIP expression and clinical features of patients, such as age, presence of metastasis and follow-up data, independently of the histological type (p>0.05, Table 2).

**Table 2:** Correlations between RKIP expression and cervical cancer patient’s clinical data.

			RKIP Expression		
Parameter		N	Negative (%)	Positive (%)	*p*
**Age (years)**					
	>49	15	12 (80)	3 (20)	0.805
	≤49	18	15 (83.3)	3 (16.7)	
**Metastasis** *					
	Absent	48	42 (87.5)	6 (12.5)	0.728
	Lymph node	5	4 (80.0)	1 (20.0)	
	Distant	14	13 (92.9)	1 (7.1)	
**Follow-up (months ± SD)**		72	123.6±6.8	95.4±10.9	0.938*

* Log-rank test determined by Kaplan-Meier method.

### RKIP Expression and Modulation of ERK Pathway in Cervical Cancer Cell Lines

**Figure 2:**
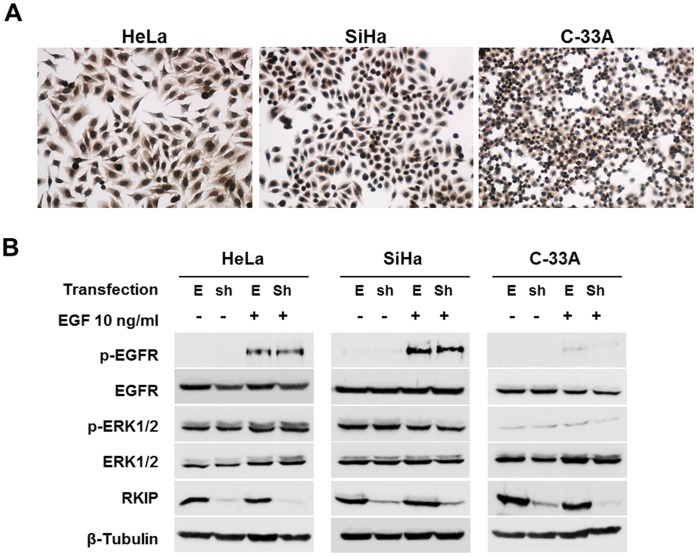
Effect of RKIP on ERK pathway activation in HeLa cells. **A)** Immunocytochemistry analysis for RKIP in HeLa, SiHa and C-33A cells showing both nuclear and cytoplasmatic expression. **B)** For RKIP inhibition, the cell lines were stably transfected with a shRNA for RKIP and with the respective empty vector for control. RKIP expression levels were assessed by western blot. Further, the cells were stimulated with 10 ng/ml of EGF by 10 minutes and EGFR and ERK activation (phosphorylation) was assessed by western blot for phospho-ERK1/2 and phospho-EGFR expression, but no significant differences were observed. E: Empty vector; Sh: ShRKIP.

To study the role of RKIP in cervical cancer, we first screened for RKIP expression in human cervical cancer cell lines (HeLa, SiHa and C-33A) by immunocytochemistry and western blot (Fig. 2). We found that RKIP is expressed at high levels in all cell lines, and is present at both cytoplasm and nucleus of the cells (Fig. 2A), as already described before [37]. All the cell lines were used to knockdown the expression of RKIP by stable transfection with PQY15 vector containing a short hairpin-RNA for RKIP (shRKIP). The empty vector was also transfected as control. The RKIP protein levels in these stable transfected cells were determined by western blot analysis. As shown in Fig. 2B, RKIP protein levels was efficiently inhibited in the shRKIP transfected cells when compared to the empty vector transfected cells.

To explore the role of RKIP in the modulation of EGF stimulated ERK signaling in cervical cancer, the transfected cells were stimulated with EGF, and EGFR and ERK phosphorylation levels were assessed by western blot. As it can be observed in Fig. 2B, EGF stimulation did not result in significant effects in the activation of ERK signaling in these cells, independently of RKIP status.

### Role of RKIP Expression in Cervical Cancer Cell Lines Biological Behavior

**Figure 3:**
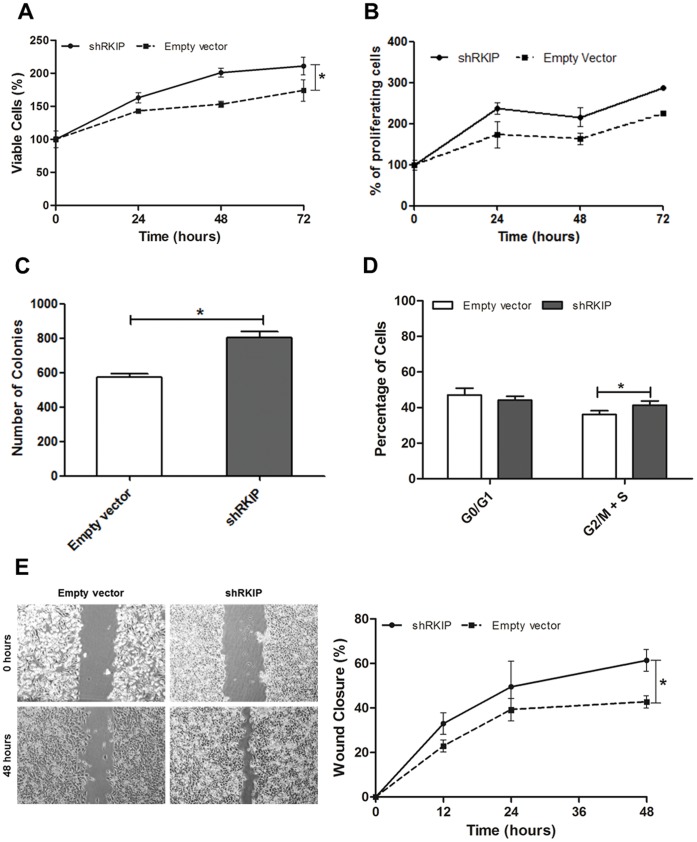
*In vitro* role of RKIP in HeLa cells biological behavior. **A)** Cellular viability and **B)** Proliferation was measured at 24, 48 and 72 hours by MTS and BrdU assays, respectively. RKIP inhibited cells had a statistically significant viability and proliferative advantage over time, when compared to control cells. **C)** Cells were assayed for their ability to proliferate in growth medium containing 0.35% agar and the formation of multi-cellular colonies photographed at x16 magnification after 14 days. RKIP inhibition induces a statistically significant anchorage-independent growth of HeLa cells in soft agar. **D)** Downregulation of RKIP in HeLa cells induced a shift on cell cycle distribution with a statistically significant higher percentage of cells in G2M+S phase when compared to control cells. Cell cycle analysis was done at 24 hours’ time point by flow cytometric analysis of propridium iodide stained cells. **E)** A standardized scratch (wound) was applied to monolayers and digital images were taken at several time points (0; 12; 24 and 48 hours). It was observed that shRKIP transfected cells had a statistically significant migration advantage over time, when compared to control cells (right panel). Representative images at 0 and 48 hours are presented in the left panel. All the experiments were done in triplicate at least three times. Data is represented as the mean ± SD and differences with a *p*<0.05 on the two-way ANOVA or student’s t test were considered statistically significant (*).

To assess whether the modulation of RKIP expression influenced the tumorigenic properties of the cells, we chose the cell line with highest level of RKIP inhibition by shRKIP, HeLa and measured *in vitro* its viability, proliferation, anchorage independent growth and migration capabilities (Fig. 3). We observed that shRKIP transfected cells have a significant viability advantage over time (Fig. 3A), when compared with the empty vector transfected cells (p<0.05). This difference may be a reflection of an increased cell number or it may reflect increased cellular metabolism. To see whether this viability advantage was due to higher proliferation rates, we studied the effect of RKIP on BrdU incorporation, cell cycle distribution and anchorage-independent growth. The BrdU assay, confirmed that shRKIP transfected cells have a significant proliferative advantage over time (Fig. 3B). The soft agar colony formation assay demonstrated a significant (p<0.05) increase in the number of the colonies formed in the shRKIP transfected cells when compared to the control cells (Fig. 3C). By cell cycle analysis, we observed that shRKIP transfected cells had a decreased G0/G1 phase with a concomitant and significant (p<0.05) increase in the G2/M+S phase (Fig. 3D). These results were further validated in the other two transfected cell lines, SiHa and C-33A (Fig. S1A–D).

Finally, to address the effect of RKIP in HeLa cells migration, we performed wound healing migration assay and observed that shRKIP transfected cells had a migration advantage over time, with a significant (p<0.05) higher rate of wound closure when compared with the control cells (Fig. 3E). The same was observed in the other two transfected cell lines, SiHa and C-33A (Fig. S1E–F).

### *In vivo* Role of RKIP Expression in Cervical Cancer Growth and Angiogenesis

**Figure 4:**
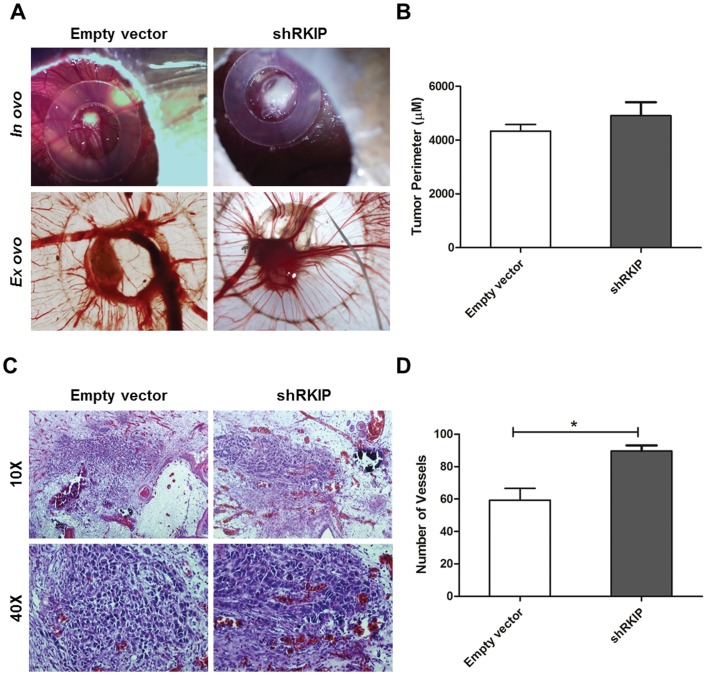
*In vivo* role of RKIP in HeLa cells growth and angiogenesis. **A)** Representative pictures (16× magnification) of CAM assay after 7 days of tumor growth *in ovo* and *ex ovo*. **B)** Tumor growth was measured *in vivo* by CAM assay as described in materials and methods section. It was observed a higher perimeter (µM) of the tumors (*in ovo*) formed by shRKIP cells, however the difference between the control cells was not significant. **C)** Hematoxylin-eosin staining of the paraffin embedded tumors showing the higher vascularization induced by RKIP inhibition. Representative pictures with 10× and 20× magnification are represented in the left panel. **D)** Counting of the blood vessels *ex ovo*, it was observed a statistically significant increase on the number of vessels recruited in the tumors formed by shRKIP cells when compared to the control. In total it was analyzed 20 eggs (10 were injected with empty vector and 10 with shRKIP cells). The data is represented as the mean ± SD and differences with a *p*<0.05 on the Student’s t test were considered statistically significant (*).

To examine the effect of RKIP-mediated tumor growth and angiogenesis *in vivo*, we performed the CAM assay (Fig. 4). The transfected cells were implanted into the CAM of the chick embryo (empty vector cells, n = 10; shRKIP cells, n = 10), and seven days after cell implantation, the chicken embryos were sacrificed to evaluate tumor growth and angiogenesis, as described in materials and methods section. The mean perimeter of the tumors formed by the control and the shRKIP transfected cells was 4335±823 µm and 4913±1568 µm, respectively. As shown in Fig. 4B, the size of the tumors formed by shRKIP cells was not significantly higher when compared to the tumors formed by the empty vector cells.

To evaluate the impact of RKIP in angiogenesis modulation, we counted *ex ovo* the number of vessels around the tumors. We counted a mean of 54±16 and 83±10 vessels in the tumors formed by the empty vector and the shRKIP transfected cells, respectively. RKIP inhibited cells revealed a statistically significant (*p*<0.05) increase in blood vessels recruitment when compared to the control cells (Fig. 4D). As shown in Fig. 4C, the hematoxylin-eosin staining of the paraffin embedded tumors and CAM’s confirmed the rich vascularization of the tumors with capillaries around the tumor and capillaries sprouting out of the CAM into the tumors. Higher rates of vascularization were observed in the tumors formed by shRKIP transfected cells (Fig. 4C).

### Role of RKIP in Cervical Cancer Cells Response to Chemotherapy

**Figure 5:**
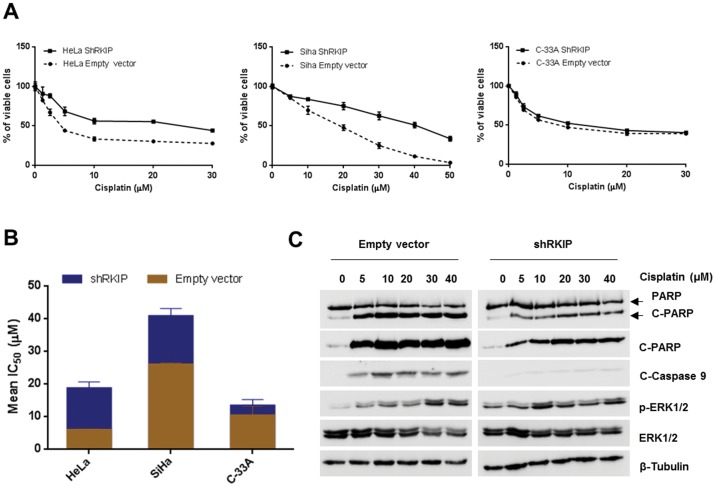
Role of RKIP in cervical cancer cells response to cisplatin. **A)** Representative pictures of the nonlinear regression analysis of cervical cancer cell lines treated with cisplatin for determination of half maximal inhibitory concentrations (IC_50_). **B)** Graphic representation of the mean IC_50_ values for cisplatin in the cervical cancer cell lines. The transfected cells with shRKIP were less sensitive to cisplatin treatment in the three different cell lines. **C)** HeLa transfected cell lines were exposed to increasing concentrations of cisplatin by 24 hours. Cisplatin treatment induced ERK activation (p-ERK1/2) and apoptosis of the cells as evaluated by PARP (total and cleaded specific antibodies) and caspase-9 cleavage, mainly in the empty vector transfected cells. All the experiments were done in triplicate at least three times. Data in the panels A and B are represented as the mean ± SD.

To determine whether RKIP protein could be involved in the modulation of cervical cancer patient’s response to the conventional chemotherapy, we determined the sensibility of cervical cancer cell lines transfected with shRKIP to cisplatin treatment. As it can be observed in the cytotoxic assays (Fig. 5A), all the shRKIP transfected cell lines were less sensitive to cisplatin treatment, when compared to the control cell lines transfected with the empty vector, being this difference less evident for C-33A cell line. As shown in Fig. 5B, HeLa cells transfected with the shRKIP displayed a mean IC_50_ of 18.55±2.06 µM against the 5.91±0.30 µM reached by the empty vector cells. For C-33A cell line the values were more similar between the two transfected cell lines, 10.37±2.69 µM and 13.25±1.98 µM for the empty vector and the shRKIP transfected cells, respectively (Fig. 5B). The SiHa cell line was the less sensitive to cisplatin with the empty vector transfected cells reaching 25.99±0.51 µM of mean IC_50_, while the shRKIP transfected cells reached 40.69±2.37 µM of mean IC_50_ for cisplatin (Fig. 5B).

To explore the mechanisms by which RKIP induces resistance to cisplatin in cervical cancer cell lines, we exposed HeLa cells to increasing concentrations of cisplatin during 24 hours (Fig. 5C), and found that cisplatin treatment induced ERK activation in both cell lines, more evident for higher doses, but shRKIP transfected cells were more resistant to cisplatin induced PARP and capase-9 cleavage (Fig. 5C). Thus, RKIP seems to modulate cervical cancer cells response to cisplatin chemotherapy by controlling apoptosis.

## Discussion

The role played by HPV in cervical cancer initiation is well known; however, the molecular pathways involved in the progression and metastasization processes remains unclear [6]. While the broad use of Papanicolaou smear screening has led to a decline in mortality from cervical cancer, many patients still died of the disease, mainly due to cancer metastasis [44]. Therefore, it is important to elucidate the mechanisms involved in cervical cancer aggressiveness and metastisation.

In cancer, RKIP is considered to be a metastasis suppressor gene, being downregulated during the metastatic process of distinct tumors [18], [20]–[23]. In the present study, we studied the protein expression levels of RKIP not only in cervical cancer, but also in non-malignant lesions (cervicitis and intraepithelial lesions). In general, we found that RKIP is highly expressed at the cytoplasm in the benign lesions (54% of the samples; 73/135), but is significantly (*p*<0.05) reduced in cervical cancer (independently of the histological subtype), with only ∼15% (19/124) of the samples staining positively. No associations were found between RKIP expression and the presence of metastasis. Also, no correlations were found between the remaining patient’s clinicopathological data or survival, which limits its use as prognostic marker. However, further studies, involving a larger number of patients with a more comprehensive clinicopathological data available, are needed to validate and extend our findings. Additionally, it would be interesting to analyze the interplay between RKIP and HPV infection. Hu CJ and colleagues have recently reported results very similar to ours: the high expression of RKIP in non-tumoral or pre-malignant lesions, and its significant lost in primary tumors of the cervix and in its lymph node metastases. Similar to us, the authors did not found significant correlation between RKIP positivity and clinical outcome [36]. In many other studies, loss of RKIP protein has been associated with poor prognosis [18], [22], [25]–[28], [30], [33], [34], but at variance, many studies, including ours, have come out showing the absence of RKIP in primary tumors independently of associations with presence of tumor metastasis or prognosis [10], [11], [24], [28], [31], [32], [45], [46].

RKIP action was first described as an inhibitor of the RAF-1- MEK interaction, thereby preventing the activation of RAF/MEK/ERK signal transduction [8]. Recently, several studies have showed that RKIP is involved in more intracellular signaling pathways. Stimulation of cells with a growth factor, such as EGF, induces RKIP phosphorylation at S153 by protein kinase C reduces the affinity of RKIP for c-RAF and enhances RKIP binding to G-protein–coupled receptor kinase-2 [7], [47]. Moreover, RKIP suppresses the activation of the nuclear factor Kappa B (NFkB) cell survival pathway by blocking the inactivation of the inhibitor of NFkB, namely IkB [48]. More recently, it was shown that RKIP binds GSK3 proteins and maintains GSK3β protein levels and its active form [49]. The involvement of RKIP in signal transduction pathways that are important regulators of growth, motility, invasion and differentiation in cancer cells, re-enforce the usefulness of functional studies to assess its role in tumors where its expression is downregulated.

To understand the biological role of the reduction of RKIP in cervical cancer specimens, we performed *in vitro* and *in vivo* studies with three distinct cervical cancer cell lines. RKIP downregulation was done by cells transfection with a specific shRNA for RKIP, which resulted in the reduction of the protein levels, as already described [37], [50].

Firstly, we studied the role of RKIP in the control of cervical cancer cells growth. We observed that cells with low levels of RKIP had *in vitro* a viability advantage over time when compared with cells with normal levels of RKIP. This viability advantage was shown to be due to the high levels of proliferation of the cells, as evidenced by BrdU and colony formation assays, which is probably promoted by a shift in cell cycle distribution with a higher percentage of cells in G2/M and S phase. The *in vitro* effect of RKIP in cellular proliferation and cell cycle regulation has not been consistent in the literature, and this may reflect in part the assays used. Our data agrees with those of Li *et al* who obtained similar results in ovarian cancer cell lines with regard to viability and anchorage-independent growth [45]. Also, Lee *et al* found that the proliferation of human hepatoma cells was reduced in cells overexpressing RKIP [51]. However, Schuierer *et al* reported that RKIP did not influence melanoma cell proliferation *in vitro* [52], an observation that was also supported using prostate cells [20]. Regarding cell cycle, our results were similar to those obtained by other authors in ovarian and nasopharyngeal carcinomas [45], [53]. However, previous studies with HeLa cells showed a change in mitotic index but no significant change in cell proliferation [37].

*In vivo*, we did not observed differences in the size of the tumors formed by RKIP inhibited cells when compared to control cells, consistent with results from mouse xenograft models [19]. Dangi-Garimella *et al* demonstrated that RKIP regulates invasion, intravasation and metastasis in human breast cancer but does not significantly alter primary tumor growth *in vivo*, consistent with a role as a metastasis suppressor [19]. Thus, the discrepant *in vitro* and *in vivo* findings suggest that RKIP function can be influenced by the tumor microenvironment.

To explore the putative role of RKIP in the regulation of cervical cancer metastasis, we studied the migratory and angiogenic properties of RKIP downregulated cells. We found *in vitro* that the absence of RKIP is related with higher migratory capability of the cells, and *in vivo* that tumors formed by injection of shRKIP cells were significantly more vascularized. Similar results using wound healing migration assay were already obtained with other cell types, such as kidney, fibroblasts and hepatoma cells [12], [50], [51]. Concerning angiogenesis, our results are in agreement with the results reported by Li *et al*, who reported the low expression of RKIP associated with angiogenesis in a breast cancer mouse model [54]. Likewise, Fu *et al* reported that overexpression of RKIP reduces the angiogenic capacity and vascular invasion in a prostate mouse model [20], this being a crucial mechanism through which RKIP regulates metastasis.

Importantly, we observed a clear apoptotic resistance of cervical cancer cells to cisplatin treatment associated with low levels of RKIP expression. Other studies have shown that RKIP is also involved in the regulation of tumor cells response to both radio and chemotherapy. In drug-sensitive cell lines, down-regulation of RKIP led to the resistance to DNA-damaging drugs (9-nitrocamptothecin, taxol and cisplatin) [55]. Loss of RKIP expression in cancers leads to transcriptional activation of NFkB [48], resulting in a dramatic inhibition of apoptosis and the development of chemoresistance [56]–[58]. In nasopharyngeal carcinoma patients low expression of RKIP was associated with radiotherapy resistance [53]. Recently, Al-Mulla F et al analyzed the mechanisms through which RKIP induced chemotherapeutic resistance [59]. Using colon cancer cell lines as models, they reported a link between RKIP-KEAP 1-NRF2 to explain the drug resistance induced by RKIP and suggest this pathway as a possible target for personalized therapeutic intervention in RKIP depleted cancers [59].

Based on these findings, therapies that can induce increased RKIP expression can constitute interesting approaches. Locostatin has been shown to abrogate the ability of RKIP to inhibit Raf-1. Increasing the levels of RKIP expression re-sensitize cells to drug induced apoptosis [60]. Rituximab has also shown to up-regulate RKIP, which has been shown to sensitize non-Hodgkin’s lymphoma cell lines to chemotherapeutic induced apoptosis [61]. Furthermore, nitric oxide mediated chemo/immunosensitization via inhibition of NFkB may also involve the induction of RKIP. Induction of RKIP inhibits anti-apoptotic pathways that regulate tumor cell sensitivity to apoptotic stimuli [62].

### Conclusions

In conclusion, we confirm that RKIP protein is significantly lost in cervical carcinomas when compared with precursor lesions. Furthermore, we found that RKIP downregulation in cervical cancer cells is able to alter cell cycle distribution, induce higher cellular survival, proliferation, migration and anchorage independent growth *in vitro.* In contrast, no effects were observed in the tumors growth *in vivo*, but it was found that RKIP inhibition is associated with higher angiogenic rates in cervical cancer. Furthermore, we observed that RKIP expression has an impact on cervical cells effective response to cisplatin treatment. Altogether, our results would indicate RKIP as a potential biomarker for cervical cancer patients.

## Supporting Information

Figure S1 **In vitro role of RKIP in SiHa and C-33A cells biological behavior.** **A–B)** Cellular viability was measured by MTS. RKIP inhibited cells had a statistically significant viability advantage over time, when compared to control cells. **C–D)** Cells were assayed for their ability to proliferate in growth medium containing 0.35% agar and the formation of multi-cellular colonies photographed at x16 magnification after 14 days. RKIP inhibition induces a statistically significant anchorage-independent growth in soft agar. **E–F)** A standardized scratch (wound) was applied to monolayers and digital images were taken at several time points. It was observed that shRKIP transfected cells had a statistically significant migration advantage over time, when compared to control cells. All the experiments were done in triplicate at least three times. Data is represented as the mean ± SD and differences with a p<0.05 on the two-way ANOVA or student’s t test were considered statistically significant (*).
(TIF)

## References

[1] Jemal A, Bray F, Center MM, Ferlay J, Ward E, et al. (2011) Global cancer statistics. CA Cancer J. Clin 61: 69–90. caac.20107 [pii];10.3322/caac.20107 [doi]. 10.3322/caac.20107 21296855

[2] Castellsague X, Diaz M, de SS, Munoz N, Herrero R, et al (2006) Worldwide human papillomavirus etiology of cervical adenocarcinoma and its cofactors: implications for screening and prevention. J Natl Cancer Inst 98: 303–315. 16507827 10.1093/jnci/djj067

[3] Bosch FX, de SS (2007) The epidemiology of human papillomavirus infection and cervical cancer. Dis Markers 23: 213–227. 17627057 10.1155/2007/914823 PMC3850867

[4] zur HH (2002) Papillomaviruses and cancer: from basic studies to clinical application. Nat Rev Cancer 2: 342–350. 12044010 10.1038/nrc798

[5] Davey DD (2003) Cervical cytology classification and the Bethesda System. Cancer Journal 9: 327–334. 10.1097/00130404-200309000-00002 14690307

[6] Martin AG (2007) Molecular biology of cervical cancer. Clin Transl Oncol 9: 347–354. DOI 10.1007/s12094-007-0066-8. 10.1007/s12094-007-0066-8 17594948

[7] Corbit KC, Trakul N, Eves EM, Diaz B, Marshall M, et al.. (2003) Activation of Raf-1 signaling by protein kinase C through a mechanism involving Raf kinase inhibitory protein. J Biol Chem 278: 13061–13068. 10.1074/jbc.M210015200 [doi];M210015200 [pii]. 10.1074/jbc.M210015200 12551925

[8] Yeung K, Seitz T, Li S, Janosch P, McFerran B, et al.. (1999) Suppression of Raf-1 kinase activity and MAP kinase signalling by RKIP. Nature 401: 173–177. 10.1038/43686 [doi]. 10.1038/43686 10490027

[9] Yeung K, Janosch P, McFerran B, Rose DW, Mischak H, et al (2000) Mechanism of suppression of the Raf/MEK/extracellular signal-regulated kinase pathway by the raf kinase inhibitor protein. Mol Cell Biol 20: 3079–3085. 10757792 10.1128/mcb.20.9.3079-3085.2000 PMC85596

[10] Akaishi J, Onda M, Asaka S, Okamoto J, Miyamoto S, et al (2006) Growth-suppressive function of phosphatidylethanolamine-binding protein in anaplastic thyroid cancer. Anticancer Res 26: 4437–4442. 17201166

[11] Zhang L, Fu Z, Binkley C, Giordano T, Burant CF, et al.. (2004) Raf kinase inhibitory protein inhibits beta-cell proliferation. Surgery 136: 708–715. 10.1016/j.surg.2003.12.013 [doi];S0039606004000200 [pii]. 10.1016/j.surg.2003.12.013 15349122

[12] Al-Mulla F, Bitar MS, Taqi Z, Rath O, Kolch W (2010) RAF kinase inhibitory protein (RKIP) modulates cell cycle kinetics and motility. Mol. Biosyst 7: 928–41. 10.1039/c0mb00208a [doi]. 10.1039/c0mb00208a 21180766

[13] Bement WM (2005) A role for RKIP in cell motility. Chem Biol 12: 953–954. S1074–5521(05)00268–1 [pii];10.1016/j.chembiol.2005.08.012 [doi]. 10.1016/j.chembiol.2005.08.012 16183016

[14] Baritaki S, Chapman A, Yeung K, Spandidos DA, Palladino M, et al.. (2009) Inhibition of epithelial to mesenchymal transition in metastatic prostate cancer cells by the novel proteasome inhibitor, NPI-0052: pivotal roles of Snail repression and RKIP induction. Oncogene 28: 3573–3585. onc2009214 [pii];10.1038/onc.2009.214 [doi]. 10.1038/onc.2009.214 19633685

[15] Hellmann J, Rommelspacher H, Muhlbauer E, Wernicke C (2010) Raf kinase inhibitor protein enhances neuronal differentiation in human SH-SY5Y cells. Dev Neurosci 32: 33–46. 000236595 [pii];10.1159/000236595 [doi]. 10.1159/000236595 19955695

[16] Keller ET, Fu Z, Brennan M (2004) The role of Raf kinase inhibitor protein (RKIP) in health and disease. Biochem Pharmacol 68: 1049–1053. 10.1016/j.bcp.2004.04.024 [doi];S0006295204003600 [pii]. 10.1016/j.bcp.2004.04.024 15313400

[17] Keller ET, Fu Z, Yeung K, Brennan M (2004) Raf kinase inhibitor protein: a prostate cancer metastasis suppressor gene. Cancer Lett 207: 131–137. 15151133 10.1016/j.canlet.2004.02.006

[18] Martinho O, Simões K, Longatto-Filho A, Jacob CE, Zilberstein B, et al.. (2013) Absence of RKIP expression is an independent prognostic biomarker in gastric cancer. Oncol Rep 29(2): 690–6. 10.3892/or.2012.2179 [doi]. 10.3892/or.2012.2179 23232914

[19] Dangi-Garimella S, Yun J, Eves EM, Newman M, Erkeland SJ, et al.. (2009) Raf kinase inhibitory protein suppresses a metastasis signalling cascade involving LIN28 and let-7. EMBO J 28: 347–358. emboj2008294 [pii];10.1038/emboj.2008.294 [doi]. 10.1038/emboj.2008.294 PMC2646152 19153603

[20] Fu Z, Smith PC, Zhang L, Rubin MA, Dunn RL, et al (2003) Effects of raf kinase inhibitor protein expression on suppression of prostate cancer metastasis. J Natl Cancer Inst 95: 878–889. 12813171 10.1093/jnci/95.12.878

[21] Hagan S, Al-Mulla F, Mallon E, Oien K, Ferrier R, et al.. (2005) Reduction of Raf-1 kinase inhibitor protein expression correlates with breast cancer metastasis. Clin Cancer Res 11: 7392–7397. 11/20/7392 [pii];10.1158/1078–0432.CCR-05–0283 [doi]. 10.1158/1078-0432.CCR-05-0283 16243812

[22] Al-Mulla F, Hagan S, Behbehani AI, Bitar MS, George SS, et al (2006) Raf kinase inhibitor protein expression in a survival analysis of colorectal cancer patients. J Clin Oncol. 24: 5672–5679. 10.1200/JCO.2006.07.5499 17179102

[23] Minoo P, Zlobec I, Baker K, Tornillo L, Terracciano L, et al (2007) Loss of Raf-1 kinase inhibitor protein expression is associated with tumor progression and metastasis in colorectal cancer. Am J Clin Pathol. 127: 820–827. 10.1309/5D7MM22DAVGDT1R8 17439843

[24] Kim HS, Kim GY, Lim SJ, Kim YW (2010) Raf-1 kinase inhibitory protein expression in thyroid carcinomas. Endocr Pathol 21: 253–257. 10.1007/s12022-010-9131-x [doi]. 10.1007/s12022-010-9131-x 20734161

[25] Martinho O, Gouveia A, Silva P, Pimenta A, Reis RM, et al.. (2009) Loss of RKIP expression is associated with poor survival in GISTs. Virchows Arch 455: 277–284. 10.1007/s00428-009-0821-z [doi]. 10.1007/s00428-009-0821-z 19705153

[26] Maresch J, Birner P, Zakharinov M, Toumangelova-Uzeir K, Natchev S, et al.. (2010) Additive effect on survival of Raf kinase inhibitor protein and signal transducer and activator of transcription 3 in high-grade glioma. Cancer. 10.1002/cncr.25799 [doi]. 10.1002/cncr.25799 24048798

[27] Kim HS, Kim GY, Lim SJ, Kim YW (2010) Loss of Raf-1 kinase inhibitory protein in pancreatic ductal adenocarcinoma. Pathology 42: 655–660. 10.3109/00313025.2010.522172 [doi]. 10.3109/00313025.2010.522172 21080875

[28] Xu YF, Yi Y, Qiu SJ, Gao Q, Li YW, et al.. (2010) PEBP1 downregulation is associated to poor prognosis in HCC related to hepatitis B infection. J Hepatol 53: 872–879. S0168–8278(10)00618–5 [pii];10.1016/j.jhep.2010.05.019 [doi]. 10.1016/j.jhep.2010.05.019 20739083

[29] Zaravinos A, Chatziioannou M, Lambrou GI, Boulalas I, Delakas D, et al.. (2010) Implication of RAF and RKIP Genes in Urinary Bladder Cancer. Pathol. Oncol. Res. 17: 181–90. 10.1007/s12253–010–9295–1 [doi]. 10.1007/s12253-010-9295-1 20853079

[30] Chatterjee D, Sabo E, Tavares R, Resnick MB (2008) Inverse association between Raf Kinase Inhibitory Protein and signal transducers and activators of transcription 3 expression in gastric adenocarcinoma patients: implications for clinical outcome. Clin Cancer Res 14: 2994–3001. 14/10/2994 [pii];10.1158/1078–0432.CCR-07–4496 [doi]. 10.1158/1078-0432.CCR-07-4496 18483365

[31] Houben R, Michel B, Vetter-Kauczok CS, Pfohler C, Laetsch B, et al.. (2006) Absence of classical MAP kinase pathway signalling in Merkel cell carcinoma. J Invest Dermatol 126: 1135–1142. 5700170 [pii];10.1038/sj.jid.5700170 [doi]. 10.1038/sj.jid.5700170 16498399

[32] Chen Y, Ouyang GL, Yi H, Li MY, Zhang PF, et al (2008) Identification of RKIP as an invasion suppressor protein in nasopharyngeal carcinoma by proteomic analysis. J Proteome Res 7: 5254–5262. 19367706 10.1021/pr800602c

[33] Zlobec I, Baker K, Minoo P, Jass JR, Terracciano L, et al (2008) Node-negative colorectal cancer at high risk of distant metastasis identified by combined analysis of lymph node status, vascular invasion, and raf-1 kinase inhibitor protein expression. Clin Cancer Res 14: 143–148. 18172264 10.1158/1078-0432.CCR-07-1380

[34] Fu Z, Kitagawa Y, Shen R, Shah R, Mehra R, et al.. (2006) Metastasis suppressor gene Raf kinase inhibitor protein (RKIP) is a novel prognostic marker in prostate cancer. Prostate 66: 248–256. 10.1002/pros.20319 [doi]. 10.1002/pros.20319 16175585

[35] Biewenga P, Buist MR, Moerland PD, van Thernaat EVL, van Kampen AHC, et al.. (2008) Gene expression in early stage cervical cancer. Gynecologic Oncology 108: 520–526. DOI 10.1016/j.ygyno.2007.11.024. 10.1016/j.ygyno.2007.11.024 18191186

[36] Hu CJ, Zhou L, Zhang J, Huang C, Zhang GM (2011) Immunohistochemical detection of Raf kinase inhibitor protein in normal cervical tissue and cervical cancer tissue. J Int Med Res 39: 229–237. 21672326 10.1177/147323001103900125

[37] Eves EM, Shapiro P, Naik K, Klein UR, Trakul N, et al.. (2006) Raf kinase inhibitory protein regulates aurora B kinase and the spindle checkpoint. Mol Cell 23: 561–574. S1097–2765(06)00496–5 [pii];10.1016/j.molcel.2006.07.015 [doi]. 10.1016/j.molcel.2006.07.015 PMC1626587 16916643

[38] Logarinho E, Maffini S, Barisic M, Marques A, Toso A, et al.. (2012) CLASPs prevent irreversible multipolarity by ensuring spindle-pole resistance to traction forces during chromosome alignment. Nat Cell Biol 14: 295–303. ncb2423 [pii];10.1038/ncb2423 [doi]. 10.1038/ncb2423 22307330

[39] Cardeal LB, Boccardo E, Termini L, Rabachini T, Andreoli MA, et al.. (2012) HPV16 oncoproteins induce MMPs/RECK-TIMP-2 imbalance in primary keratinocytes: possible implications in cervical carcinogenesis. PLoS One 7: e33585. 10.1371/journal.pone.0033585 [doi];PONE-D-12–01424 [pii]. 10.1371/journal.pone.0033585 PMC3306414 22438955

[40] Martinho O, Faloppa CC, Scapulatempo NC, Longatto-Filho A, Baiocchi G, et al.. (2012) Loss of RKIP expression during the carcinogenic evolution of endometrial cancer. J. Clin. Pathol. 65: 122–8. jclinpath-2011–200358 [pii];10.1136/jclinpath-2011–200358 [doi]. 10.1136/jclinpath-2011-200358 22031589

[41] Martinho O, Granja S, Jaraquemada T, Caeiro C, Miranda-Goncalves V, et al.. (2012) Downregulation of RKIP Is Associated with Poor Outcome and Malignant Progression in Gliomas. PLoS One 7: e30769. 10.1371/journal.pone.0030769 [doi];PONE-D-11–15118 [pii]. 10.1371/journal.pone.0030769 PMC3264629 22292035

[42] Trakul N, Menard RE, Schade GR, Qian Z, Rosner MR (2005) Raf kinase inhibitory protein regulates Raf-1 but not B-Raf kinase activation. J Biol Chem 280: 24931–24940. M413929200 [pii];10.1074/jbc.M413929200 [doi]. 10.1074/jbc.M413929200 15886202

[43] Moniz S, Martinho O, Pinto F, Sousa B, Loureiro C, et al (2013) Loss of WNK2 expression by promoter gene methylation occurs in adult gliomas and triggers Rac1-mediated tumour cell invasiveness. Hum Mol Genet. 22: 84–95. 10.1093/hmg/dds405 23035050

[44] Poomtavorn Y, Suwannarurk K, Thaweekul Y, Maireang K (2011) Risk Factors for High-Grade Cervical Intraepithelial Neoplasia in Patients with Atypical Squamous Cells of Undetermined Significance (ASC-US) Papanicolaou Smears. Asian Pac J Cancer Prev 12: 235–238. 21517264

[45] Li HZ, Wang Y, Gao Y, Shao J, Zhao XL, et al.. (2008) Effects of raf kinase inhibitor protein expression on metastasis and progression of human epithelial ovarian cancer. Mol Cancer Res 6: 917–928. 6/6/917 [pii];10.1158/1541–7786.MCR-08–0093 [doi]. 10.1158/1541-7786.MCR-08-0093 18567796

[46] Zaravinos A, Kanellou P, Baritaki S, Bonavida B, Spandidos DA (2009) BRAF and RKIP are significantly decreased in cutaneous squamous cell carcinoma. Cell Cycle 8: 1402–1408. 8308 [pii]. 10.4161/cc.8.9.8308 19342899

[47] Lorenz K, Lohse MJ, Quitterer U (2003) Protein kinase C switches the Raf kinase inhibitor from Raf-1 to GRK-2. Nature 426: 574–579. 10.1038/nature02158 [doi];nature02158 [pii]. 10.1038/nature02158 14654844

[48] Yeung KC, Rose DW, Dhillon AS, Yaros D, Gustafsson M, et al.. (2001) Raf kinase inhibitor protein interacts with NF-kappaB-inducing kinase and TAK1 and inhibits NF-kappaB activation. Mol Cell Biol 21: 7207–7217. 10.1128/MCB.21.21.7207–7217.2001 [doi]. 10.1128/MCB.21.21.7207-7217.2001 PMC99896 11585904

[49] Al-Mulla F, Bitar MS, Al-Maghrebi M, Behbehani AI, Al-Ali W, et al.. (2011) Raf Kinase Inhibitor Protein RKIP Enhances Signaling by Glycogen Synthase Kinase-3{beta}. Cancer Res. 15: 1334–43. 0008–5472.CAN-10–3102 [pii];10.1158/0008–5472.CAN-10–3102 [doi]. 10.1158/0008-5472.CAN-10-3102 21303975

[50] Shemon AN, Eves EM, Clark MC, Heil G, Granovsky A, et al.. (2009) Raf Kinase Inhibitory Protein protects cells against locostatin-mediated inhibition of migration. PLoS One 4: e6028. 10.1371/journal.pone.0006028 [doi]. 10.1371/journal.pone.0006028 PMC2696091 19551145

[51] Lee HC, Tian B, Sedivy JM, Wands JR, Kim M (2006) Loss of Raf kinase inhibitor protein promotes cell proliferation and migration of human hepatoma cells. Gastroenterology 131: 1208–1217. S0016–5085(06)01537-X [pii];10.1053/j.gastro.2006.07.012 [doi]. 10.1053/j.gastro.2006.07.012 PMC2593881 17030190

[52] Schuierer MM, Bataille F, Hagan S, Kolch W, Bosserhoff AK (2004) Reduction in Raf kinase inhibitor protein expression is associated with increased Ras-extracellular signal-regulated kinase signaling in melanoma cell lines. Cancer Res 64: 5186–5192. 10.1158/0008–5472.CAN-03–3861 [doi];64/15/5186 [pii]. 10.1158/0008-5472.CAN-03-3861 15289323

[53] Ruan L, Wang GL, Yi H, Chen Y, Tang CE, et al.. (2010) Raf kinase inhibitor protein correlates with sensitivity of nasopharyngeal carcinoma to radiotherapy. J Cell Biochem 110: 975–981. 10.1002/jcb.22611 [doi]. 10.1002/jcb.22611 20564197

[54] Li HZ, Gao Y, Zhao XL, Liu YX, Sun BC, et al.. (2009) Effects of raf kinase inhibitor protein expression on metastasis and progression of human breast cancer. Mol Cancer Res 7: 832–840. 1541–7786.MCR-08–0403 [pii];10.1158/1541–7786.MCR-08–0403 [doi]. 10.1158/1541-7786.MCR-08-0403 19531568

[55] Chatterjee D, Bai Y, Wang Z, Beach S, Mott S, et al (2004) RKIP sensitizes prostate and breast cancer cells to drug-induced apoptosis. J Biol Chem 279: 17515–17523. 14766752 10.1074/jbc.M313816200

[56] Wu K, Bonavida B (2009) The activated NF-kappaB-Snail-RKIP circuitry in cancer regulates both the metastatic cascade and resistance to apoptosis by cytotoxic drugs. Crit Rev Immunol 29: 241–254. 18841dcb1a005a88,0d4f451d6774e29d [pii]. 10.1615/critrevimmunol.v29.i3.40 19538137

[57] Woods Ignatoski KM, Grewal NK, Markwart SM, Vellaichamy A, Chinnaiyan AM, et al.. (2008) Loss of Raf kinase inhibitory protein induces radioresistance in prostate cancer. Int J Radiat Oncol Biol Phys 72: 153–160. S0360–3016(08)00835–3 [pii];10.1016/j.ijrobp.2008.04.072 [doi]. 10.1016/j.ijrobp.2008.04.072 PMC2597029 18722266

[58] Chatterjee D, Bai Y, Wang Z, Beach S, Mott S, et al.. (2004) RKIP sensitizes prostate and breast cancer cells to drug-induced apoptosis. J Biol Chem 279: 17515–17523. 10.1074/jbc.M313816200 [doi];M313816200 [pii]. 10.1074/jbc.M313816200 14766752

[59] Al-Mulla F, Bitar MS, Feng J, Park S, Yeung KC (2012) A new model for raf kinase inhibitory protein induced chemotherapeutic resistance. PLoS One 7: e29532. 10.1371/journal.pone.0029532 [doi];PONE-D-11-09100 [pii]. 10.1371/journal.pone.0029532 PMC3261143 22279539

[60] Zhu S, Mc Henry KT, Lane WS, Fenteany G (2005) A chemical inhibitor reveals the role of Raf kinase inhibitor protein in cell migration. Chem Biol 12: 981–991. 16183022 10.1016/j.chembiol.2005.07.007

[61] Jazirehi AR, Vega MI, Chatterjee D, Goodglick L, Bonavida B (2004) Inhibition of the Raf-MEK1/2-ERK1/2 signaling pathway, Bcl-xL down-regulation, and chemosensitization of non-Hodgkin’s lymphoma B cells by Rituximab. Cancer Res 64: 7117–7126. 15466208 10.1158/0008-5472.CAN-03-3500

[62] Bonavida B, Baritaki S, Huerta-Yepez S, Vega MI, Chatterjee D, et al (2008) Novel therapeutic applications of nitric oxide donors in cancer: roles in chemo- and immunosensitization to apoptosis and inhibition of metastases. Nitric Oxide 19: 152–157. 18477483 10.1016/j.niox.2008.04.018

