# Supplementary figures and images for: RKIP Inhibition in Cervical Cancer Is Associated with Higher Tumor Aggressive Behavior and Resistance to Cisplatin Therapy
Source: PLoS One. 2013 Mar 19;8(3):e59104. doi: 10.1371/journal.pone.0059104 (PMC3602518; doi:10.1371/journal.pone.0059104)

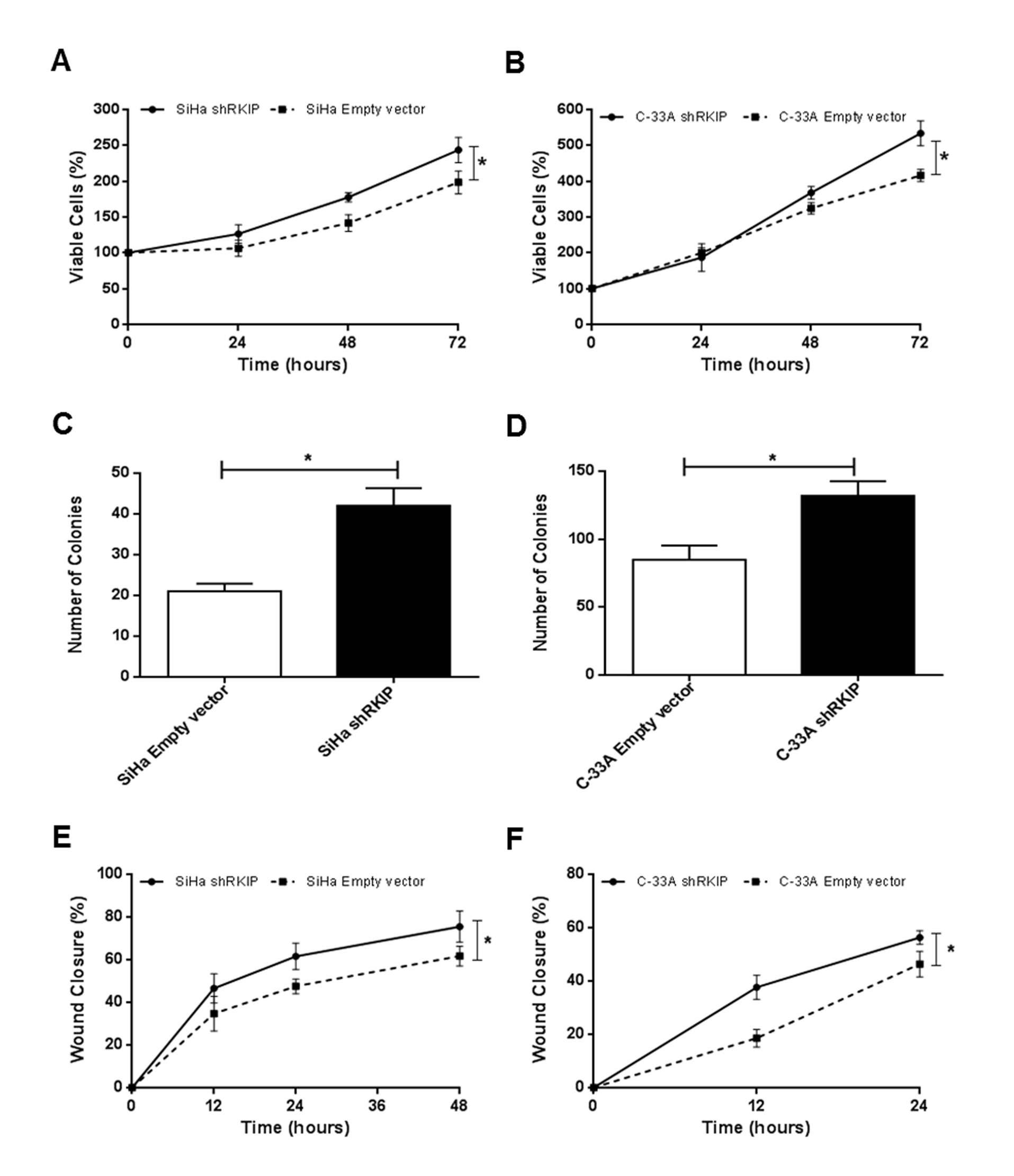

Supplement: Figure S1 — In vitro role of RKIP in SiHa and C-33A cells biological behavior. A–B) Cellular viability was measured by MTS. RKIP inhibited cells had a statistically significant viability advantage over time, when compared to control cells. C–D) Cells were assayed for their ability to proliferate in growth medium containing 0.35% agar and the formation of multi-cellular colonies photographed at x16 magnification after 14 days. RKIP inhibition induces a statistically significant anchorage-independent growth in soft agar. E–F) A standardized scratch (wound) was applied to monolayers and digital images were taken at several time points. It was observed that shRKIP transfected cells had a statistically significant migration advantage over time, when compared to control cells. All the experiments were done in triplicate at least three times. Data is represented as the mean ± SD and differences with a p<0.05 on the two-way ANOVA or student’s t test were considered statistically significant (*). (TIF) [file pone.0059104.s001.tif]
